# Journey of a Patient with CKD in India

**DOI:** 10.34067/KID.0000000000000124

**Published:** 2023-05-25

**Authors:** Vasundhara Raghavan, Urmila Anandh

**Affiliations:** 1 Kidney Warriors Foundation, Mumbai, India; 2 Department of Nephrology, Amrita Hospitals, Sector 88, Faridabad, Delhi, NCR, Pin 121002 India

**Keywords:** CKD, patient self-assessment

## Introduction

CKD affects more than 850 million people worldwide and will become the fifth largest cause of years of life lost by 2040.^1^ This CKD epidemic has caused a major economic strain not only in developing countries but also in low-income and middle-income countries. Economically advanced countries offer their citizens all aspects of renal replacement therapy, while lesser-resourced countries struggle to engage patients with appropriate treatments.^2^ This perspective highlights the challenges faced and the patients' biggest concerns in the management of CKD and is coauthored by a kidney donor (V.R.) who has walked the tortuous road of kidney disease with a family member and went on to start the largest nongovernmental organization for patients with CKD (Kidney Warriors Foundation [KWF]) in India.

## Diagnosis of CKD

CKD in India is often diagnosed late.^3^ Lack of awareness often plays an important role, and the absence of symptoms (higher incidence of interstitial nephritis and CKD of unknown origin) contributes to the delay. The burden of kidney failure is increasing, with almost 210,000 new cases being diagnosed each year.^4^

**Figure 1:**
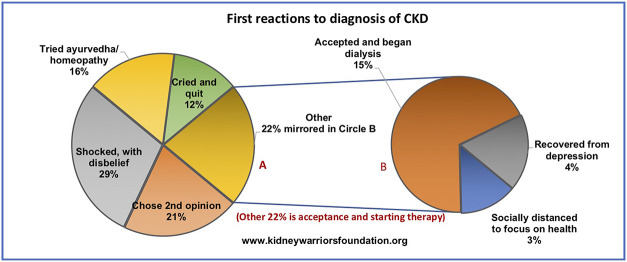
**First reactions of the patients to the diagnosis of CKD.** (The larger circle depicts all the responses, whereas the smaller circle looks at the positive responses categorized as “other” in the larger circle).

When diagnosed with CKD, most often at an advanced stage, patients initially reject their diagnosis, have survival worries, and look for an escape route. Understanding and accepting CKD has been tough for Indian patients. Through an authentic poll conducted on Facebook by KWF, the early reactions of patients were analyzed (Figure 1). As is obvious, most find it very difficult to cope.

The situation can be improved if the newly diagnosed patients get the support of counselors and experienced patients. In addition, it is imperative that we raise awareness in the population that kidney disease is not a “death sentence” as it is made out to be. As patients accept reality, they start building survival strategies.

## Therapy of CKD in India

In India, managing CKD is complicated for patients due to various socioeconomic factors. With very little financial support and a lack of nephrologists, patients find it difficult to access health care services. They often default therapy because of financial constraints and progress to end-stage kidney failure.^5^ Under pressure, many start alternative medications which often worsen their kidney disease.

When kidney replacement therapy is needed, it is often hemodialysis.

Dialysis therapy is well streamlined in many parts of India now. However, with the increasing number of patients needing dialysis, facilities are burdened, and quality gets compromised. Although the Government of India offers dialysis through various schemes, few people continue treatment because they cannot afford to miss their daily wages on their dialysis day.^6^ Peritoneal dialysis is hardly offered.

## Issues with Transplantation

A transplant is considered the best treatment, but getting a kidney transplant requires determination and dedicated effort by the patient and family. Finding a willing, suitable donor, with good histocompatibility leukocyte antigen and cross match, and getting appropriate clearances is a major challenge. Long wait time for deceased donor transplants is another reality.

The Transplantation of Human Organs and Tissues Act 1994, amended in 2011, has laid down rules and regulations. New rules to expand the “donor pool” were added to redefine “near relatives.” However, there are many challenges in optimizing renal transplantation in India. Many nongovernmental organizations have voiced their concerns and have suggested solutions to make the kidney transplantation process hassle-free and patient friendly, without compromising the basic values of organ donation (Table 1).

**Table 1:** Challenges and possible solutions for patient friendly transplantation process in India

Challenges	Possible Solutions as Suggested by Patient Bodies/Voluntary Organizations
1. All states have not adopted the 1994 Act and 2011 amendments. Many states have yet to pass the amendments. Law is a state subject.	Urgent need for a single, unified law governed by central statutes.
2. Lack of information about the transplantation procedure. Legislation and policy makers need to recognize the patient, and the team involved in transplant laws must include the patient/caregiver in the decision-making.	One consolidated document on information to be prepared by the appropriate authorities with dos and donts which will remove obscurity and individual interpretation of the law.
3. Kidney transplants are not performed in all states. Patients in states with nonexistent transplant programs are at a major disadvantage.	A system linking India uniformly as a country will remove stumbling blocks of domicile and multiple no objection certificates facilitating interstate travel for transplant treatment.
4. Long wait times for legal and other bureaucratic formalities.	Members authorized to issue approvals must be conversant with medical conditions and should have a modicum of empathy. They should prevent these vulnerable and sick patients to move from pillar to post getting their necessary approvals.
5. State financial assistance is usually restricted for patients who are getting their treatment within the state.	Assistance must not be determined by physical boundaries and must be applicable anywhere in India.
6. Unethical and illegal practices are a big dampener for genuine donors. In this chaotic situation, the authorization committee gets innovative and often stops transplants as a knee jerk reaction. In most situations, transplant activities are suspended, further aggravating the situation.	Strict legal process and punishment for miscreants will safeguard patient's and health care professional's concerns. The law should leave no ambiguity as to how to go about such situation and law enforcement agencies should act appropriately.
7. Movies and television series talk about kidney sale as a way of making a “quick buck” and dishonor sick kidney patients awaiting a transplant.	The society needs to sensitize itself that the “joking” mention of “selling a kidney” is in very poor taste. As this practice continues unabated, the government needs to come down heavily on these messages/mentions in movies, television, and social media.

## Financial Challenges Faced by the Patients

The medical expenses for a patient with CKD are huge and become a major strain on families. Doctor visits, investigations, and other recurrent costs are often difficult even for a middle-class patient. Emergency hospitalization before initiation of dialysis is common as patients are reluctant to start dialysis electively. Dialysis costs incurred by a patient in a public sector hospital are approximately Rs 2500 ($30) and may double in a corporate hospital.^7^ Added costs include fistula surgery, erythropoietin injections, travel, and medications. A large section of middle-income patients pays for their own treatment.

Although there are multiple government-based insurance schemes, they often do not support the complete therapy requirements. Some pay for a limited number of dialysis sessions (10/mo), some partially reimburse the whole cost, and some do not address the issue of medications.^4^ Most state financial support systems are inadequate and do not factor in the social burden (travel costs, loss of employment, wages, *etc.*). Studies show that more than two thirds of the patients discontinue dialysis within 1 year because of financial issues and/or death.^4^ Many patients who continue with long-term dialysis are forced to liquidate their assets. Ten years of dialysis costs approximately Rs 30 lakhs (approximately 15 years of annual *per capita* income in India).

Economic analyses have shown that transplantation is cheaper in the long run. However, for most of the patients, even the required one-time expenses are often difficult to afford.^8^ Other expenses which are often not factored in include unforeseen complications postoperatively. Certain quasireligious and nongovernmental organizations offer to cover the entire procedure cost for economically poorer patients. However, these organizations do not support maintenance immunosuppression, and this leads to situations of disastrous medication nonadherence.

## The Way Forward

Patients with a diagnosis of CKD in India often find it difficult to access and receive therapy. Despite remarkable progress in nephrology in India over the past five to six decades, there are still concerns of inclusivity and equity for many patients. Much needs to be performed to mitigate the harrowing experience of these vulnerable patients.

The first step is to include CKD as part of the public policy dialogs. Because they are not included in noncommunicable disease initiatives, patients with CKD are deprived of adequate care and almost two thirds of them die without treatment.^4^ CKD should be integrated within ongoing health programs of the government.^9^ The government is setting up a national dialysis mission in 630 districts which may to a certain extent improve the situation, but much more is needed.^4^ Not only providing dialysis is important but it should also be offered to the most vulnerable living in remote and inaccessible parts of this vast country and should be of a high quality, and societal costs must be subsidized. Major nongovernmental organizations, including KWF, are consistently engaging the government in a conversation trying to improve the lot of these patients. As a part of the national oversight committee of the national dialysis program, the KWF engages with the government functionaries bringing into focus issues which matter most to patients with CKD.

The process of kidney transplantation in India has many challenges. Because of ethical and legal issues, the actual need of an early transplant is often overshadowed. Sensitivity and understanding of a patient's fragile health instead of mocking their need for a kidney should be a message to the public. Delayed approval and lengthy processes could result in deaths and a lost opportunity for transplant if the proposed donor develops cold feet. Then, there is an acute shortage of organs. To address this issue, authorities should facilitate the deceased donor organ transplantation program (India has a predominantly living donor programs).^10^ Continuous and combined efforts of the government, citizens of India, and nongovernmental organizations should put all their efforts to increase the organ donation rate. The KWF is committed to providing all help and support to all initiatives which make the journey of a patient with CKD less challenging.
